# Morusin inhibits cell proliferation and tumor growth by down-regulating c-Myc in human gastric cancer

**DOI:** 10.18632/oncotarget.19231

**Published:** 2017-07-14

**Authors:** Feng Wang, Dunke Zhang, Jingxin Mao, Xiao-Xue Ke, Rui Zhang, Chao Yin, Ning Gao, Hongjuan Cui

**Affiliations:** ^1^ State Key Laboratory of Silkworm Genome Biology, Southwest University, Chongqing, P.R. China; ^2^ Department of Pharmacognosy, College of Pharmacy, Third Military Medical University, Chongqing, P.R. China

**Keywords:** morusin, c-Myc, cell proliferation, tumor growth, gastric cancer

## Abstract

Morusin is a pure extract from the root bark of *Morus australis* (Moraceae). In recent years, morusin has been reported to exhibit anti-tumor biological activity in some types of human cancers through different mechanisms. Here, we attempted to investigate the inhibitory effect and mechanism of morusin on gastric cancer. Morusin markedly inhibited gastric cancer cell proliferation by down-regulating CDKs and Cyclins, such as CDK2, CDK4, Cyclin D1 and Cyclin E1. Additionally, morusin suppressed tumor growth *in vitro* and *in vivo*. Up-regulation of CDKs and Cyclins in gastric cancer cells was induced by c-Myc binding at the E-Box regions of CDKs and the Cyclin promoter. In addition, compared with the control group, the morusin-treated group showed reduced expression of c-Myc and c-Myc protein binding at the E-Box regions. Based on these results, we overexpressed c-Myc in gastric cancer cells and found that overexpressing c-Myc rescued morusin-induced inhibition of cell proliferation and tumor growth. These results suggest that morusin inhibits cell proliferation and tumor growth by down-regulating c-Myc in human gastric cancer.

## INTRODUCTION

Despite the reduced incidence of gastric cancer over the past decades, it remains one of the most common cancers worldwide [1, 2]. Gastric cancer is currently the third leading cause of cancer-related deaths in developing countries [3, 4] and accounts for nearly 100,000 new cases each year [3, 4]. Although tremendous advances have been made in therapies to treat gastric cancer, including surgery, radiation and chemotherapy, overall clinical outcomes in affected patients remain poor, with a 5-year survival rate lower than 30% [2, 5–7]. Therefore, identifying efficient crude drugs and developing chemo-preventive agents that can serve as alternative strategic options are crucial for improving gastric cancer outcomes.

Previous studies have shown that flavonoids, including flavone, flavanone, isoflavone, isoflavanone, and dihydrochalcone, have some anti-tumor activity. For example, baicalein is derived from a flavonoid that possesses diverse biological properties and has been reported to induce apoptosis in bladder cancer and inhibit proliferation in non-small cell lung cancer both *in vitro* and *in vivo* [8–10]. Chalcone, a flavonoid precursor, suppresses gastric cancer cell growth by inactivating NF-κB [11] and induces apoptosis in human colon cancer and hepatocellular carcinoma cells by up-regulating DR5 and DR4 [12–14].

Morusin, a prenylated flavonoid that was isolated from the root bark of *Morus australis* (*Moraceae*), has been reported to exhibit anti-tumor biological activity in some types of human cancers [15]. Notably, in human colorectal cancer and cervical cancer, morusin induced apoptosis and suppressed the activity of NF-κB [16, 17]. Morusin inhibited glioblastoma cell growth by regulating EGFR and DR5 to induce TRAIL sensitization both *in vivo* and *in vitro* [18, 19], suppressed breast cancer cell growth via C/EBPβ- and PPARγ-mediated lipoapoptosis [20], and induced cell death by inactivating STAT3 signaling in prostate cancer cells [21].

In light of these previous finding, we hypothesized that morusin might inhibit cell proliferation and tumor growth in human gastric cancer. However, this question has not been previously investigated. This study is the first to demonstrate that morusin possesses anti-tumor activity in gastric cancer cells. Our results suggest that morusin, a natural compound, might be a therapeutic option or alternative strategic option for gastric cancer patients.

## RESULTS

### Morusin inhibits gastric cancer cell growth and proliferation

**Figure 1:**
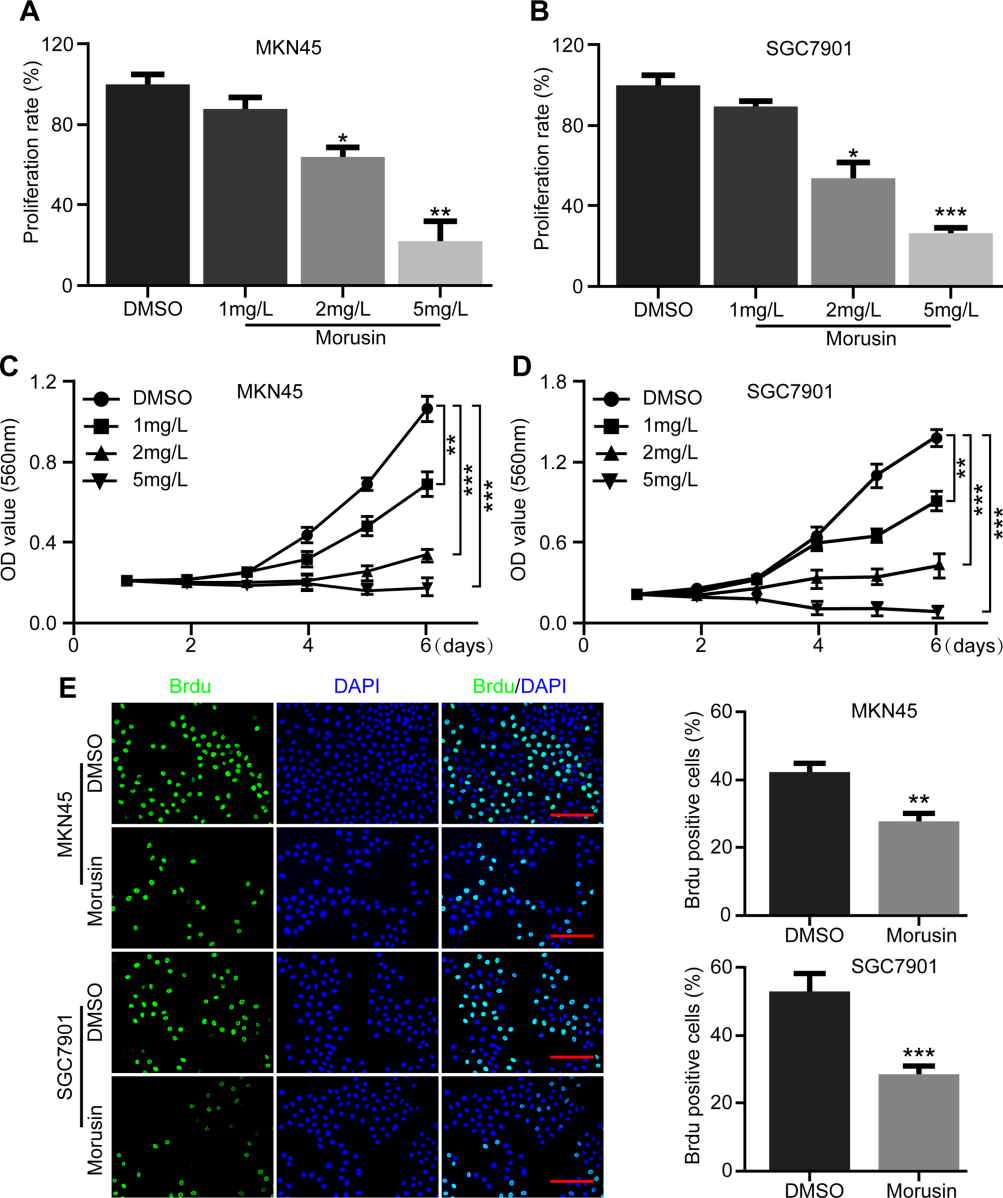
Morusin inhibits gastric cancer cell growth and proliferation (**A**, **B**) Cell numbers were counted after morusin treatment at the indicated concentration for 72 h. The histogram demonstrates the quantification of proliferation rate. DMSO was used as the control. (**C**, **D**) Cell growth was monitored using MTT assays in cells treated with morusin at the indicated times and concentrations. (**E**) Immunofluorescence staining for BrdU was performed. DAPI was used for nuclear staining. Scale bar, 20 μm. The histogram demonstrates the quantification of the rate of BrdU-positive cells. All data were analyzed using 2-tailed Student’s tests. Error bars, *P < 0.05, **P < 0.01, and ***P < 0.001.

In this study, gastric cancer cell lines (MKN45 and SGC7901) were treated with different concentrations of morusin for 72 h. The results of cell counting and proliferation rate assays indicated that morusin efficiently inhibited gastric cancer cell proliferation in a dose-dependent manner (Figure 1A and 1B). To confirm this result, MTT assays were performed to demonstrate that gastric cancer cell proliferation was significantly inhibited by morusin, as shown in Figure 1C and 1D. Moreover, the percent of BrdU-positive cells was lower after cells were treated with 2 mg/L morusin for 72 h in both cell lines (Figure 1E). In particular, morusin-treated SGC7901 cells exhibited more than a 50% reduction in BrdU-positive cells. These results demonstrate that morusin dramatically inhibited cell growth and proliferation in human gastric cancer cells.

### Morusin inhibits cell growth by inducing cell cycle arrest at the G1 phase

**Figure 2:**
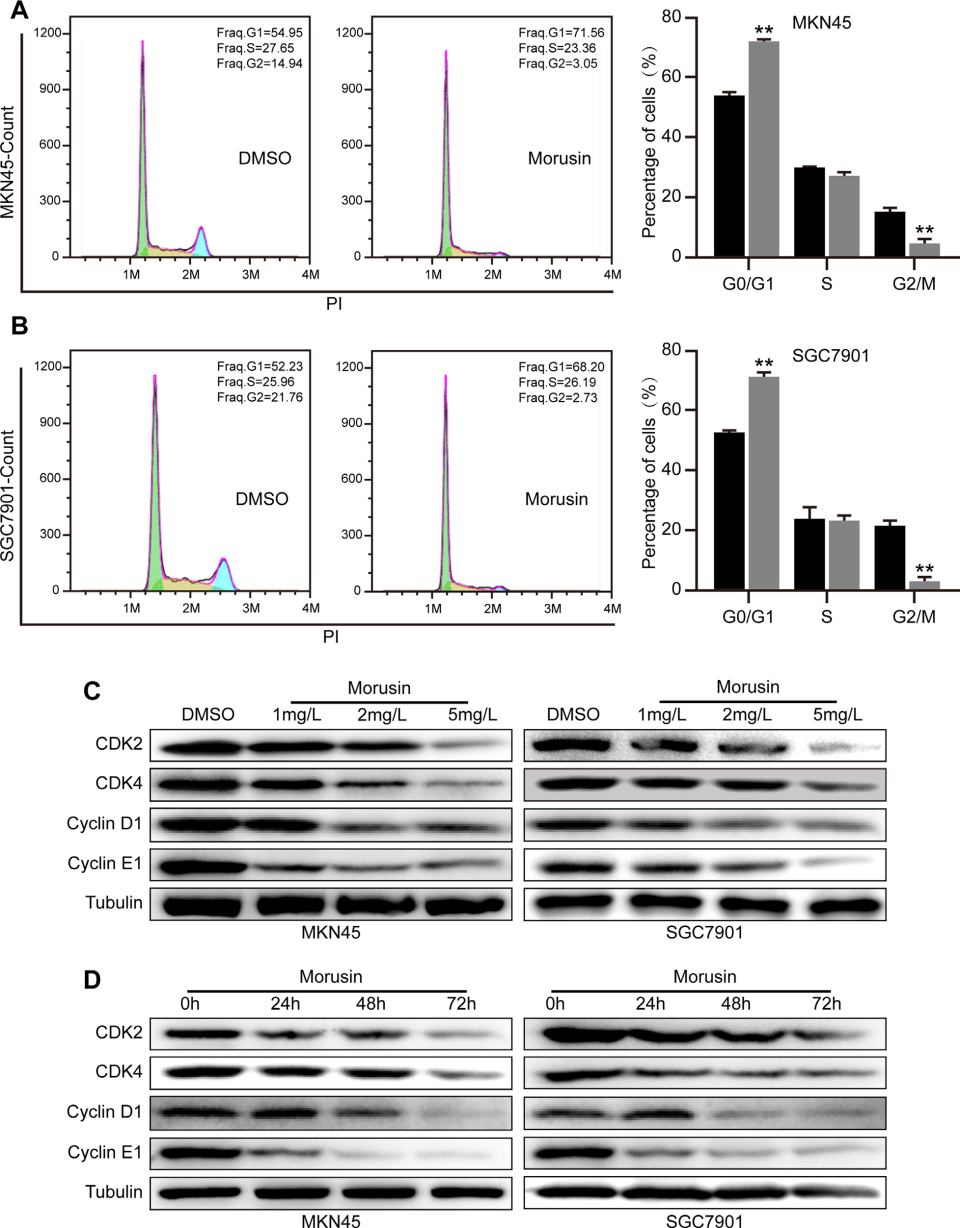
Morusin inhibits cell growth by inducing cell cycle arrest at the G1 phase (**A**, **B**) Cell cycle analyses were performed using flow cytometry in morusin-treated MKN45 (A) and SGC7901 (B) cells. The histogram demonstrates the quantification of the number of cells in different periods. (**C**, **D**) The expression levels of CDK2, CDK4, Cyclin D1 and Cyclin E1 were determined using western blot analysis after cells were treated with morusin at different concentrations (C) or at different times (D). Tubulin was used as a loading control. All data were analyzed using 2-tailed Student’s tests. Error bars, *P < 0.05, **P < 0.01, and ***P < 0.001.

To gain insight into the mechanisms underlying the ability of morusin to inhibit gastric cancer cells, we performed cell cycle assays and flow cytometry in cells treated with 2 mg/L morusin for 72 h. A major result of these experiments was that morusin arrested gastric cancer cells at the G1 phase, as shown in Figure 2A and 2B. The number of cells in the G1 phase increased by approximately 20% when both cell lines were treated with morusin. To determine the molecular mechanism underlying morusin-mediated cell cycle arrest, we performed western blot assays and found that the levels of CDKs and Cyclins, which play central roles in cell cycle progress, were decreased in morusin-treated cells in a dose- and time-dependent manner (Figure 2C, 2D and Supplementary Figure 1A). The qRT-PCR data supported a similar conclusion (Supplementary Figure 1B). These results suggest that morusin induced cell cycle arrest at the G1 phase by inhibiting the expression of CDKs and Cyclins.

### Morusin suppresses tumor growth *in vitro* and *in vivo*

**Figure 3:**
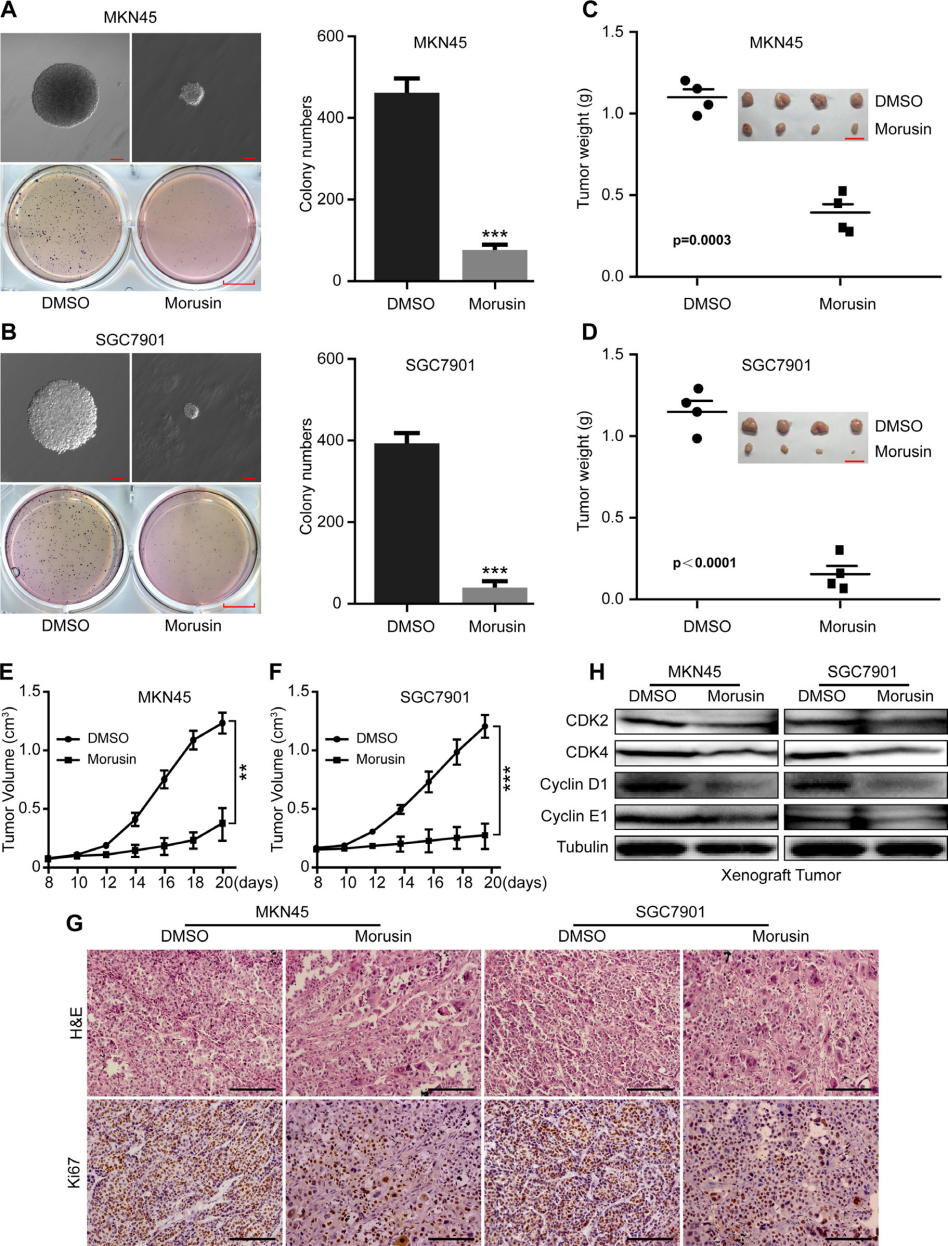
Morusin suppresses tumor growth *in vitro* and *in vivo* (**A**, **B**) Soft agar assays were performed, and the results were quantitated in morusin-treated MKN45 (A) and SGC7901 (B) gastric cancer cells. Colony formation was observed using microscopy. Scale bar: up, 100 μm; down, 25 mm. (**C**, **D**) Tumor volume and weight were measured in morusin-treated cells. Scale bar: 2 cm. (**E**, **F**) Tumor growth curves were calculated using a caliper every two days. (**G**) Hematoxylin and eosin (H&E) staining and immunohistochemical staining for Ki67 were performed. Scale bar: 50 μm. (**H**) The expression levels of the cell cycle-related proteins CDK2, CDK4, Cyclin D1 and Cyclin E1 were analyzed using western blot assays in xenograft tumors. Tubulin was used as a loading control. All data were analyzed using 2-tailed Student’s tests. Error bars, *P < 0.05, **P < 0.01, and ***P < 0.001.

To investigate the effect of morusin on tumor growth in gastric cancer cells, we evaluated tumor growth in morusin-treated cells. *In vitro*, we found that fewer and smaller colonies were produced in the morusin-treated group (2 mg/L) than in the DMSO-treated group (Figure 3A and 3B). *In vivo*, tumor volumes and weights were markedly smaller in the morusin-treated group (*P* < 0.001) than in the controls (Figure 3C and 3D), and tumor growth was significantly inhibited by morusin (Figure 3E and 3F). No obvious difference in mouse body weight was observed following treatment with morusin (Supplementary Figure 2), indicating that morusin has no influence on mouse body weight. IHC staining demonstrated that dramatically fewer cells expressed the cell proliferation marker Ki67 in the morusin-treated group (Figure 3G and Supplementary Figure 3). Additionally, we detected the levels of cell cycle-related proteins in xenograft tumors and found that the expression levels of CDK2, CDK4, Cyclin D1 and Cyclin E1 were lower in the morusin-treated group (Figure 3H). These data show that morusin-mediated inhibition of tumor growth resulted from an increase cell cycle arrest.

### c-Myc is required for morusin-induced inhibition of proliferation in gastric cancer cells

**Figure 4:**
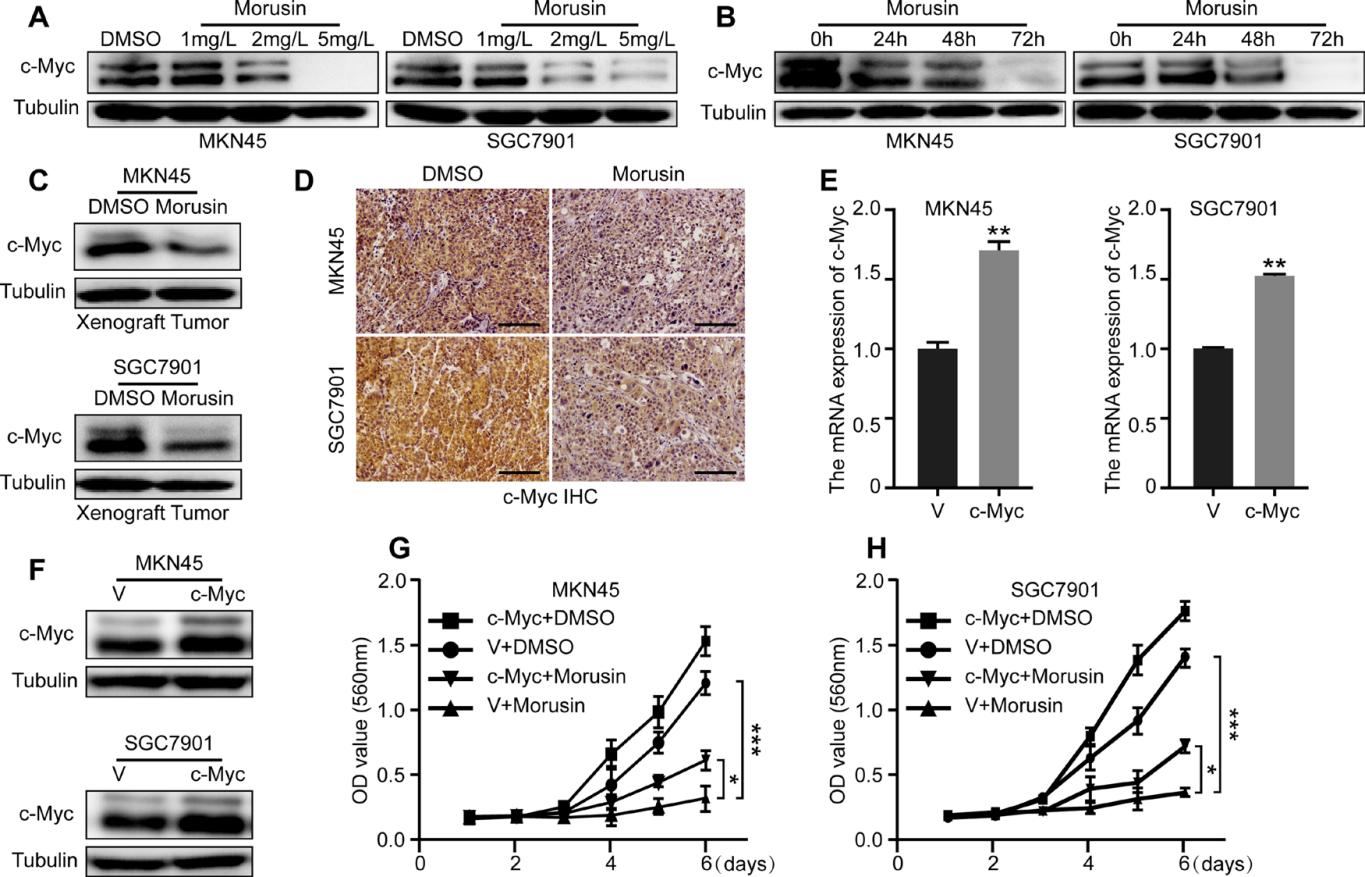
c-Myc is required for morusin-induced inhibition of proliferation in gastric cancer cells (**A**, **B**) c-Myc expression was analyzed using western blot assays after cells were treated with morusin. (**C**, **D**) c-Myc expression was analyzed using western blot assays and immunohistochemical staining in xenograft tumors. Scale bar: 50 μm. (**E**, **F**) c-Myc overexpression was evaluated using qRT-PCR and western blot assays. Tubulin was used as a loading control. (**G**, **H**) Cell growth was monitored using MTT assays in c-Myc-overexpressing cells. All data were analyzed using 2-tailed Student’s tests. Error bars, *P < 0.05, **P < 0.01, and ***P < 0.001.

The qRT-PCR and western blot assays indicated that c-Myc was gradually down-regulated in a concentration- or time- dependent manner (Figure 4A, 4B and Supplementary Figure 4A) in morusin-treated gastric cancer cells. The xenograft tumor data supported a similar conclusion (Figure 4C, 4D and Supplementary Figure 4B). In addition, we explored the underlying mechanism of reduced c-Myc and found that morusin could promote c-Myc degradation (Supplementary Figure 4C). Next, we sought to determine whether overexpressing c-Myc would rescue morusin-induced inhibition of cell growth. As shown in Figure 4E and 4F, we successfully induced c-Myc overexpression in MKN45 and SGC7901 gastric cancer cells. Cell growth was markedly higher in c-Myc overexpressing cells that were treated with morusin (*P* < 0.05) (Figure 4G and 4H), indicating that c-Myc rescued and is required for morusin-induced inhibition of cell growth in gastric cancer cells.

### c-Myc up-regulates the expression levels of CDKs and Cyclins by binding to their promoter regions in gastric cancer cells

**Figure 5:**
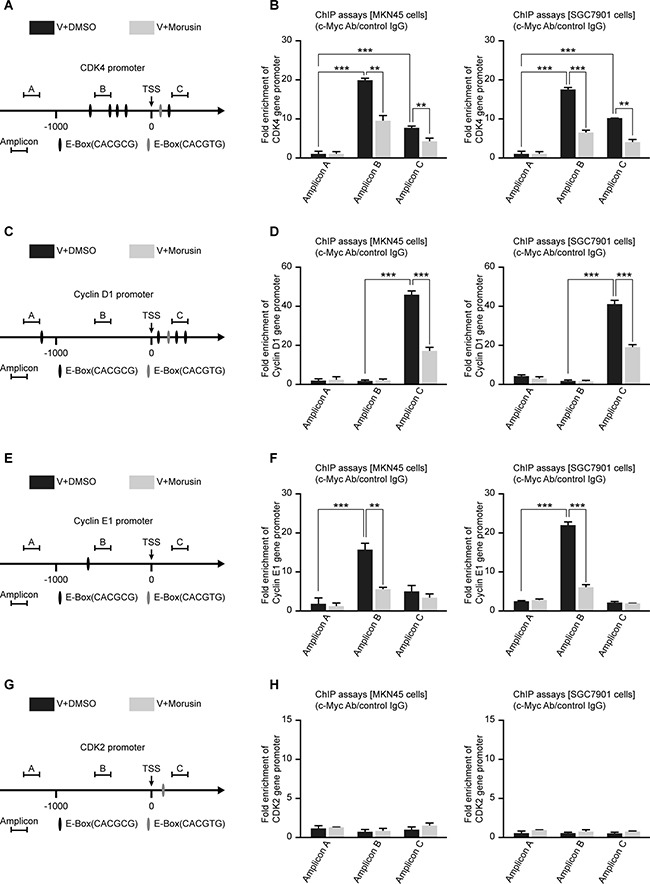
c-Myc up-regulates the expression levels of CDKs and Cyclins by binding to their promoter regions in gastric cancer cells (**A**, **C**, **E**, **G**) Schematic representation of the CDK2, CDK4, Cyclin D1 and Cyclin E1 promoters, as indicated. TSS, transcription start site. (**B**, **D**, **F**, **H**) ChIP assays were performed using MKN45 and SGC7901 cells and a C-Myc antibody, followed by qRT-PCR using primers targeting the CDK2, CDK4, Cyclin D1 and Cyclin E1 promoters, as indicated. Rabbit IgG was used as the negative control. All data were analyzed using 2-tailed Student’s tests. Error bars, *P < 0.05, **P < 0.01, and ***P < 0.001.

c-Myc up-regulates gene expression by binding to “E-box” motifs (CACGTG or CACGCG) in target gene promoters. Therefore, we analyzed the promoters of CDK2, CDK4, Cyclin D1 and Cyclin E1 and identified some “E-box” motifs at their promoter regions (Figure 5A, 5C, 5E and 5G). To confirm whether c-Myc up-regulates the expression of CDKs and Cyclins by binding to their promoters, we performed chromatin immunoprecipitation (ChIP) assays using gastric cancer MKN45 and SGC7901 cells. The ChIP assays indicated that c-Myc significantly binds to the CDK4, Cyclin D1 and Cyclin E1 gene promoter regions containing E-boxes (Figure 5B, 5D and 5F). Unfortunately, despite the E-box in the CDK2 gene promoter, we did not observe direct binding of c-Myc to the CDK2 gene promoter (Figure 5H). In addition, the ChIP assays also suggested that the morusin-treated group exhibited reduced c-Myc protein binding at the E-Box regions (Figure 5B, 5D, 5F and 5H).

After c-Myc overexpression, the mRNA expression and protein expression levels of CDKs and Cyclins were increased to varying degrees (Supplementary Figure 5A and 5B). Because RNA Pol II can promote the synthesis of mRNA precursors, we performed ChIP assays using a RNA Pol II antibody and found that c-Myc overexpression increased binding of RNA Pol II to these genes (Supplementary Figure 6). In general, our data indicate that c-Myc up-regulates the expression levels of CDKs and Cyclins by binding to their promoter regions in gastric cancer cells.

### Overexpressing c-Myc rescues morusin-induced cell cycle arrest in gastric cancer cells

**Figure 6:**
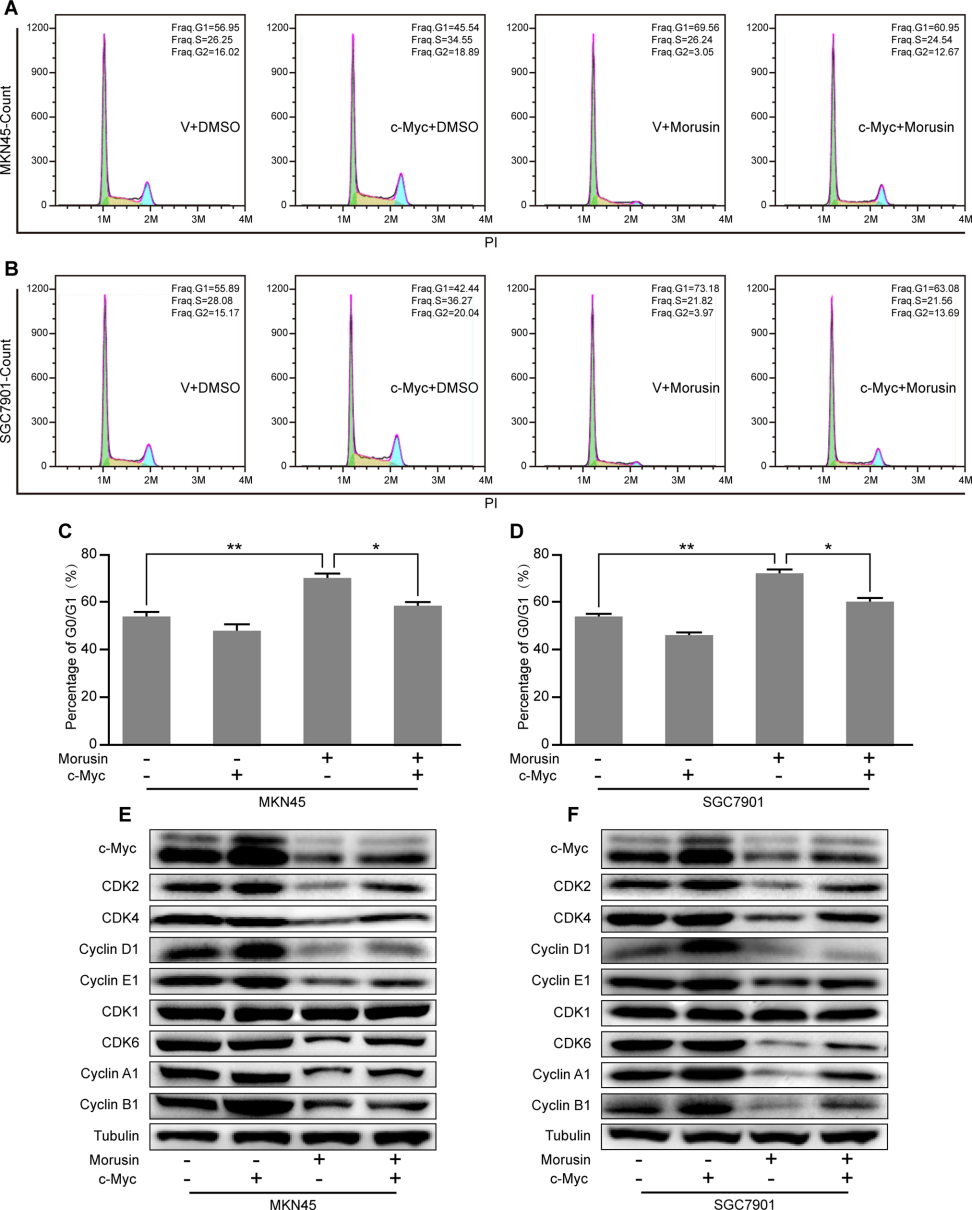
Overexpressing c-Myc rescues morusin-induced cell cycle arrest in gastric cancer cells (**A**, **B**) Cell cycle assays were performed using flow cytometry in c-Myc-overexpressing and control cells that were treated with morusin, as indicated. (**C**, **D**) A histogram demonstrates the results of quantification of the number of cells in the G1 phase. (**E**, **F**) The expression levels of cell cycle-related proteins were analyzed using western blot assays in c-Myc-overexpressing and control cells that were treated with morusin. Tubulin was used as a loading control. All data were analyzed using 2-tailed Student’s tests. Error bars, *P < 0.05, **P < 0.01, and ***P < 0.001.

Based on our previous results, we hypothesized that morusin might arrest the cell cycle by down-regulating c-Myc expression in gastric cancer cells. To confirm this hypothesis, cell cycle assays were performed in c-Myc-overexpressing and control cells that were treated with morusin (Figure 6A and 6B). As shown in Figure 6C and 6D, quantification of the flow cytometry data showed that overexpression of c-Myc decreased the number of cells in the G1 phase, even in cells that were treated with morusin. Western blot assays indicated that the expression levels of CDKs and Cyclins were higher in morusin-treated c-Myc overexpressing cells than in morusin-treated controls (Figure 6E and 6F). These data demonstrate that c-Myc overexpression rescues morusin-induced cell cycle arrest in gastric cancer cells.

### Morusin inhibits tumor growth by down-regulating c-Myc expression *in vitro* and *in* *vivo*

**Figure 7:**
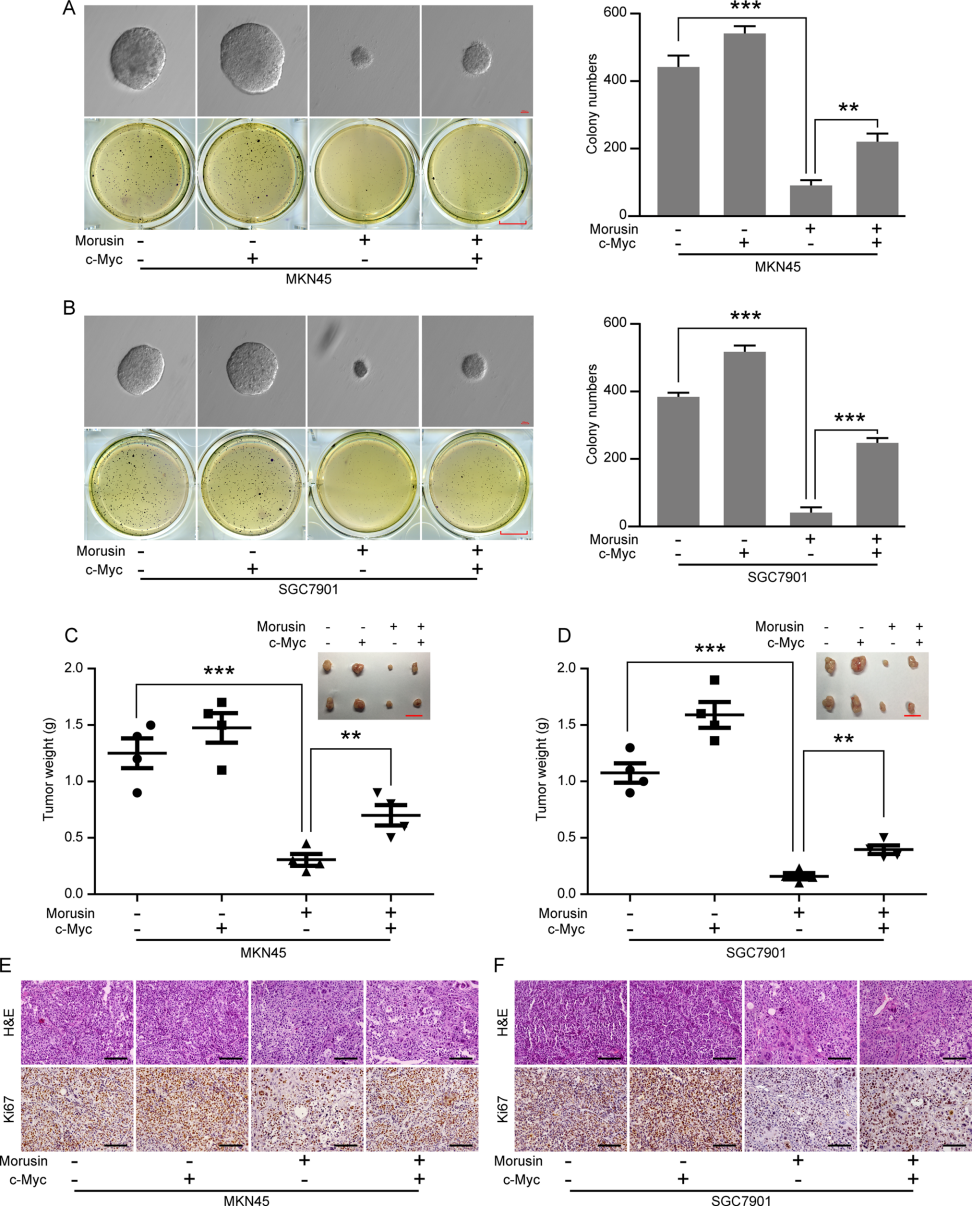
Morusin inhibits tumor growth by down-regulating c-Myc expression *in vitro* and *in vivo* (**A**, **B**) Soft agar assays were performed using c-Myc-overexpressing and control cells following treatment with morusin. Scale bar: up, 100 μm; down, 25 mm. Colony formation was quantitated using Student’s *t*-test. (**C**, **D**) Tumor volume and weight were measured in the c-Myc-overexpressing and control groups. Scale bar: 2 cm. (**E**, **F**) Hematoxylin and eosin (H&E) staining and immunohistochemical staining for Ki67 were performed. Scale bar: 50 μm. All data were analyzed using 2-tailed Student’s tests. Error bars, *P < 0.05, **P < 0.01, and ***P < 0.001.

Based on our findings that morusin inhibits c-Myc expression in a concentration- or time- dependent manner (Figure 4A and 4B) and because c-Myc is an oncogene that has been shown to promote tumor genesis and tumor growth, we next examined tumor growth ability *in vitro* and *in* *vivo* in morusin-treated c-Myc-overexpressing cells. As shown in Figure 7A and 7B, following treatment with morusin, more clones were produced in the c-Myc-overexpressing group than in the controls (Figure 7C and 7D). IHC staining indicated that the number of cells expressing the cell proliferation marker Ki67 was dramatically higher in the c-Myc-overexpressing group (Figure 7E, 7F and Supplementary 7), even after morusin treatment. These data demonstrate that morusin inhibits tumor growth by down-regulating c-Myc expression both *in vitro* and *in vivo*.

## DISCUSSION

Despite the reduced incidence of gastric cancer over the past decades, it remains the third leading cause of cancer-related deaths [3, 4]. Most gastric cancers are either asymptomatic or manifest with non-specific symptoms in early stages, which is one of the main reasons underlying delayed gastric cancer diagnoses [22]. Gastric cancer can metastasize to the lymph nodes in its early stages, representing an important prognostic factor [23]. Therefore, identifying efficient crude drugs and developing chemo-preventive agents that can serve as alternative strategic options are crucial for treating gastric cancer.

Morusin is a prenylated flavonoid that was isolated from the root bark of *Morus australis* (*Moraceae*) [15, 24, 25]. In some types of human cancers, morusin reportedly possesses anti-cancer biological activity. In this study, we treated gastric cancer cell lines with different concentrations of morusin. Our MTT and BrdU assays indicated that morusin inhibited gastric cancer cell proliferation in a dose-dependent manner. Previous reports have shown that morusin inhibits cervical cancer cell and glioblastoma cancer cell proliferation by suppressing NF-κB activity and inducing apoptosis [17, 18]. Investigations into the therapeutic effect of morusin in glioblastoma also indicated that morusin induces apoptosis and differentiation [19, 20]. In prostate cancer cells, morusin suppressed cell viability and induced apoptosis by suppressing the STAT3 pathway [26]. Based on these varying results, we performed cell cycle and western blot assays and gained insight into the underlying mechanisms of the inhibitory effect of morusin on gastric cancer cells. The major novel findings from these studies were that morusin arrested gastric cancer cells in the G1 phase by down-regulating CDKs and Cyclins.

Although many studies have demonstrated that morusin suppresses cancer cell growth and proliferation, they have not explored the effect of morusin on tumor growth. Therefore, we performed soft agar assays, which suggested that morusin inhibited tumor growth *in vitro*. *In vivo*, xenograft tumor assays indicated that morusin reduced the volume and weight of gastric cancer tumor masses by down-regulating cell cycle-related proteins and Ki67.

Interestingly, in two cell lines (MKN45 and SGC7901) treated with morusin, the expression of the proto-oncogene c-Myc was markedly decreased in both a dose- and time-dependent manner at both the mRNA and protein levels. The same result was obtained from observations of tumor tissues. Approximately 30 years ago, c-Myc was first identified as a cellular homolog of the retroviral v-Myc oncogene [27–29]. c-Myc expression is strictly regulated during normal cellular proliferation but is often deregulated in human cancers. Over 40% of gastric cancers overexpress c-Myc, which often drives poor survival [30]. As a transcription factor, c-Myc has been shown to regulate a staggering number of genes that are associated with metabolism, ribosomes, biogenesis, protein synthesis, and mitochondrial functions [31]. The activation of c-Myc is correlated with changes in the expression levels of CDKs and Cyclins; these cell cycle-related genes were the first-described “c-Myc target genes” [32]. Based on these findings, we hypothesized that morusin inhibits the expression levels of CDKs and Cyclins by down-regulating c-Myc. To confirm this hypothesis, we overexpressed c-Myc in MKN45 and SGC7901 cells. MTT assays indicated that overexpressing c-Myc partially rescued morusin-mediated inhibition of cell growth and proliferation (*P* < 0.05).

Investigations of how c-Myc regulates cell cycle-related proteins have produced conflicting results. To explore the relationship between c-Myc with CDKs and Cyclins in gastric cancer cells, we performed western blot assays. The western blot assays demonstrated that the expression levels of CDK2, CDK4, Cyclin D1 and Cyclin E1 were increased when c-Myc was overexpressed. In addition, we identified “E-box” motifs (CACGTG or CACGCG) that c-Myc could bind to in the CDK2, CDK4, Cyclin D1 and Cyclin E1 gene promoters. Based on our results, we confirmed that c-Myc binding to E-boxes regulates the expression of CDK4, Cyclin D1 and Cyclin E1 by performing ChIP assays; these assays indicated that c-Myc directly recognized and bound “E-box” motifs in their promoters. In addition, the ChIP assays also suggested that the morusin-treated group had reduced c-Myc protein binding at the E-Box regions. Regarding the relationship between CDK4, Cyclin E1 and c-Myc, our results were similar to those reported in the literature; c-Myc has been reported to directly regulate CDK4 by binding to the CDK4 promoter [33] and to up-regulate Cyclin E1 expression and induce the activation of the CDK2/Cyclin E1 complex [34]. However, the role of c-Myc in Cyclin D1 regulation is dependent on the cell type [35–37], whereas other studies have reported that c-Myc suppressed Cyclin D1 expression in BALB/c-3T3 mouse fibroblasts [38]. Our results indicated that c-Myc up-regulated Cyclin D1 expression by binding to the Cyclin D1 promoter in gastric cancer cells.

While c-Myc decreases CDK2 expression in human lung cells [39], c-Myc has no effect on CDK2 expression in other models [40]. Our data demonstrated that c-Myc up-regulated CDK2 expression, but the mechanism by which c-Myc up-regulated CDK2 expression in gastric cancer cells remains unclear.

In addition, cell cycle analyses revealed that c-Myc overexpression rescued morusin-induced inhibition of cell proliferation (*P* < 0.05). Moreover, western blot assays showed that the expression levels of CDKs and Cyclins were markedly higher when c-Myc was overexpressed in morusin-treated cells. The soft agar and xenograft tumor assays demonstrated that c-Myc overexpression rescued morusin-mediated inhibition of tumor growth both *in vitro* and *in vivo*.

In summary, the results presented here demonstrate that morusin inhibits proliferation and tumor growth by down-regulating c-Myc in gastric cancer and might be a potential neoadjuvant chemotherapy or an alternative strategy for treating gastric cancer patients.

## MATERIALS AND METHODS

### Cell culture and drug treatment

Cell lines (MKN45 and SGC7901) of human gastric cancer were obtained from American Type Culture Collection (ATCC, Rockville, MD, USA). Both of gastric cancer cell lines were cultured with 10% fetal bovine serum (FBS) and 1% penicillin-streptomycin (P/S) in Roswell Park Memorial Institute-1640 (RPMI-1640, Gibco). Cells were cultured with 5% CO_2_ at 37°C in humidified incubator. Morusin was dissolved in Dimethyl Sulfoxide (DMSO) as 2 mg/ml stock solutions. Gastric cancer cells were treated with morusin in different time or concentration.

### Lentivirus production and cell infection

We cloned human full length c-Myc cDNA (obtained from NCBI, NM_002467.4) into PCDH-CMV-GFP-MCS-EF1-puro vector. According to transfection’s instructions, lipofectamine 2000 (11668-019, Invitrogen) was used and recombinant PCDH-CMV-MCS-EF1-puro-c-Myc plasmid was co-transfecting 293FT cell line with packaging plasmids pLP1, pLP2, and pLP/VSVG for lentivirus production. For cell infection, medium containing with virus was harvested after 48 h culture.

### Cell proliferation assay

The 3-(4, 5-dimethylthiazol-2-yl)-2, 5-diphenyltetrazolium bromide (MTT) was used to obtain cell growth curves. Briefly, 1000 gastric cancer cells were plated with morusin or DMSO in 96-well plate. After 24 h culture, 20 μl MTT (5 mg/ml, Sigma) were added to each well at the indicated time points. After incubated at 37°C for 2 h, removed the medium and replaced by 200 μl DMSO, and we obtained absorbance at 560 nm using a Thermo Scientific Microplate Reader.

### Cell cycle assay

For cell cycle assay, after treated with 2 mg morusin or DMSO for 72 h. After washing with phosphate buffer solution (PBS) for 3 times, 1 × 10^6^ cells were harvested and suspended in 75% ethanol for 24 h at 4°C. Then cells were washed with PBS for 3 times, and incubated with propidium iodide (PI) (BD Biosciences, USA) containing with RNase at 37°C in dark for 1h. According to the manufacturer’s directions, Flow Cytometer (FACS C6, BD Biosciences) was used for cell cycle data analyzed.

### Western blot assay

After treated with different concentration gradient or time gradient of morusin (DMSO was used as control), we harvested all group cells and lysed with RIPA lysis buffer (Abcam, ab156034), then centrifuged (10000 g, 15 min) and separated the supernatants. 10% SDS-PAGE was used to separate lysated proteins and transferred to PVDF membrane. PVDF membrane containing total proteins was blocking with 5% defatted milk for 2 h, then incubated with primary antibody against C-myc (1:1000 Abcam ab32072), CDK1 (1:2000 Abcam ab18), CDK2 (1:1000, Cell Signaling Technology 9932T), CDK4 (1:1000, Cell Signaling Technology 9932T), CDK6 (1:1000, Cell Signaling Technology 13331T), Cyclin A1 (1:1000, Abcam ab53699), Cyclin B1 (1:1000, Cell Signaling Technology 12231T), Cyclin D1 (1:1000, Cell Signaling Technology 9932T), Cyclin E1 (1:1000, Cell Signaling Technology 9932T), Tubulin (1:2000, Enogene E12-043-03) at 4°C overnight. After washing with PBS for 3 times, PVDF membrane were incubated with secondary antibody HRP-labeled goat anti-mouse IgG (H+L) (A0216, 1:10000) or goat anti-rabbit IgG (H+L) (1:10000, A0208) at room temperature for 2 h. The signal was captured by ECL reagent (Beyotime) and visualized by Western blotting detection instruments (Clinx Science).

### Immunohistochemistry staining

Tumor specimen was embedded in paraffin and sectioned at 5 μm. Samples were incubated with rabbit C-myc primary antibody (1:100, ab32072, Abcam), or rabbit Ki67 primary antibody (1:100, 550609, BD Biosciences) at 4°C overnight. Then incubated with HRP-conjugated secondary antibodies (Cell Signaling Technology) at room temperature for 2 h. The signal was visualized with DAB reagent, and examined under a light microscopy.

### Quantitative real-time PCR (qRT-PCR)

Briefly, cells were lysed with trizol. Then RNA was extracted according to the manufacturer’s directions provided by Life Technology and cDNA was obtained. Quantitative real-time PCR (qRT-PCR) assay was accomplished, as previously described [41]. C-myc primers was used as below: F: 5′-ACAGCCCACTGGTCCTCAAG-3′; R: 5′-TCGGTTG TTGCTGATCTGTCTC-3′).

### Soft agar colony formation assay

Through soft agar assay, self-renewal ability and tumor growth *in vitro* was determined with morusin treatment. For base agar, 1 ml RPMI 1640 complete medium containing 0.6% low-gelling temperature agarose were added to 6-well plate. After solidify of 0.6% base agar, another 1 ml RPMI 1640 complete medium containing 0.3% low-gelling temperature agarose with 2 mg/L morusin were mixed with 1 × 10^3^ cells and added as top agar. Colonies were photographed after 14 to 21 days’ culture with 5% CO_2_ at 37°C in humidified incubator and recorded.

### Tumor xenograft experiment

Nude mice (BALA/c) were purchased from Beijing laboratory animal research center China. 4 weeks old mouse was used, and housed for 1 week in SPF environment before injection. 1 × 10^6^ MKN45 or SGC7901 cells in 200 ul PBS were subcutaneously injected into both flanks of each mouse respectively. One group was injected intraperitoneal with moursin at 50 mg/kg mouse body weight every two days, it’s totally for 3 weeks. As control, DMSO was used in another group. Tumor size was measured to calculated tumor volume every two days after one week tumor growth, mice body weight was also monitored every two days. Three weeks after injected cells, mice were euthanized, and tumors were weighted and immobilized using paraformaldehyde for immunohistochemistry staining. All studies were comply with the institution of the Animal Care and Use Committee of Southwest University.

### Chromatin immunoprecipitation

According to the ChIP assay kit (17-371, Millipore) instructions, chromatin immunoprecipitation (ChIP) assay was performed. At least 1 × 10^7^ MKN45 and SGC7901 cells were cross-linked by 1% formaldehyde and lysed in ChIP lysis buffer. Chromatin was sheared using ultrasonication and incubated with C-myc primary antibody (1:50 Abcam ab32072) at 4°C overnight, rabbit IgG was used as control. Then C-myc/DNA complexes were immunoprecipitated and eluted. After reverse crosslinks of C-myc/DNA complexes, DNA was purified and gathered for qRT-PCR.

### Statistical analysis

All observations were confirmed by at least three independent experiments. Quantitative data are expressed as the mean ± standard deviation. Two-tailed Student’s *t*-test was performed for paired samples. *P* < 0.05 was considered statistically significant.

## SUPPLEMENTARY MATERIALS FIGURES AND TABLE


