# Supplementary material for: Morusin inhibits cell proliferation and tumor growth by down-regulating c-Myc in human gastric cancer
Source: Oncotarget. 2017 Jul 14;8(34):57187–200. doi: 10.18632/oncotarget.19231 (PMC5593635; doi:10.18632/oncotarget.19231)
Supplement: Supplementary file 1 [file oncotarget-08-57187-s001.pdf]

Morusin inhibits cell proliferation and tumor growth by down-regulating c-Myc in human gastric cancer

SUPPLEMENTARY MATERIALS

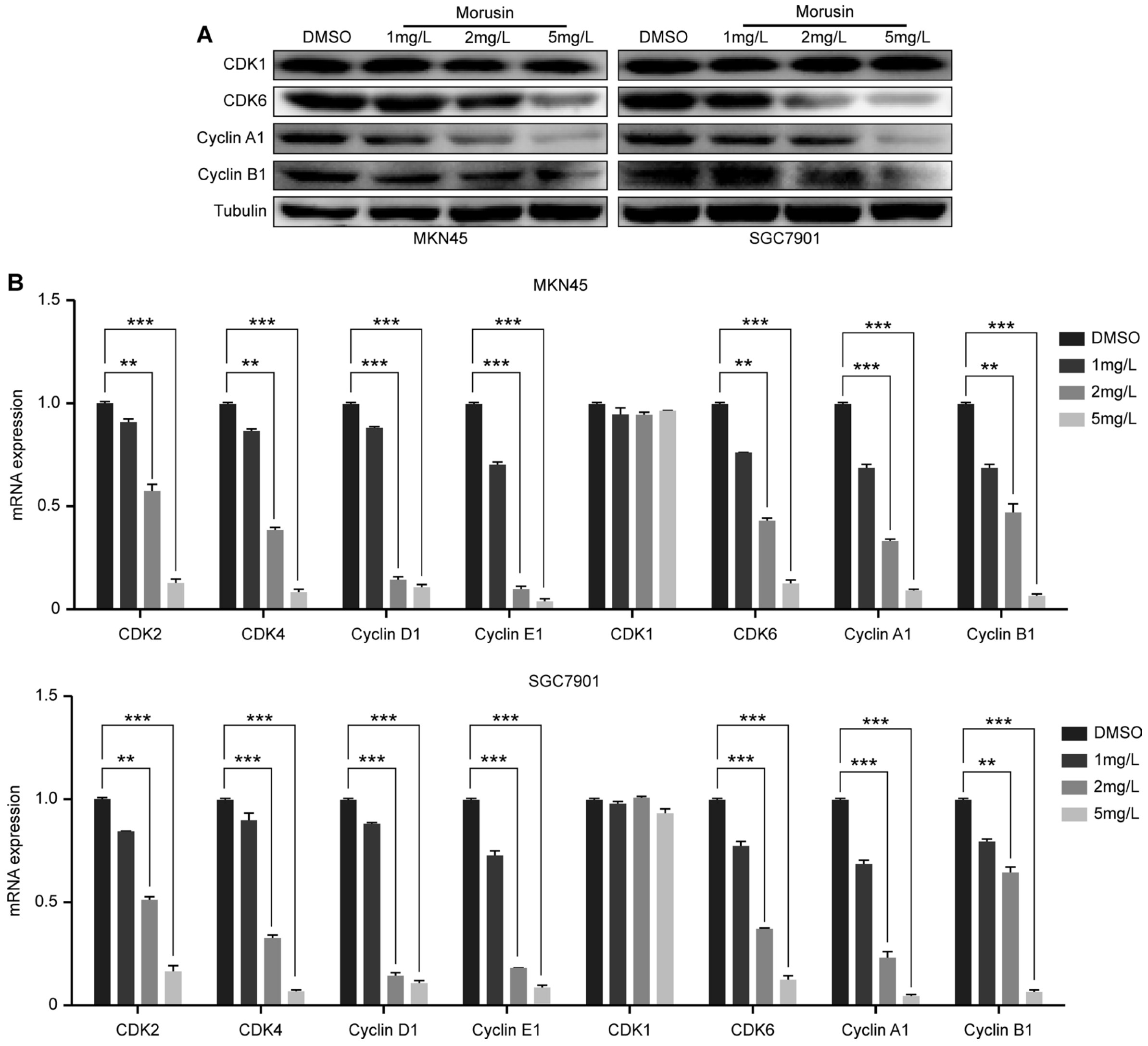

Supplementary Figure 1: Morusin down-regulates the expression of CDKs and Cyclins. (**A**, **B**) After cells were treated with morusin at different concentrations, the expression levels of CDKs and Cyclins were determined using western blot analysis (A) and qRT-PCR assays (B). All data were analyzed using 2-tailed Student's tests. Error bars, *$P < 0.05$, **$P < 0.01$, and ***$P < 0.001$.

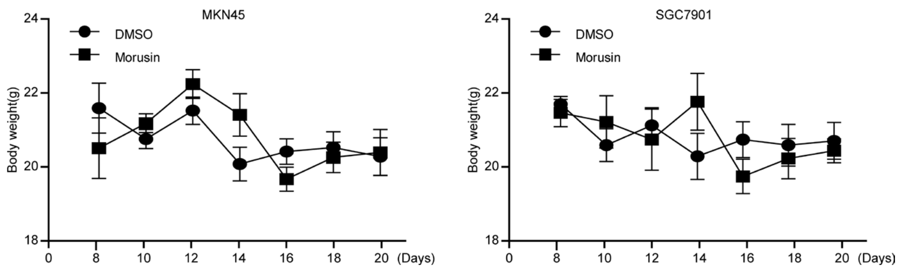

Supplementary Figure 2: Morusin has no markedly influence of the mouse weight. The weight of mouse was measured after injected with morusin or DMSO. All data were analyzed using 2-tailed Student's tests. Error bars, *$P < 0.05$, **$P < 0.01$, and ***$P < 0.001$.

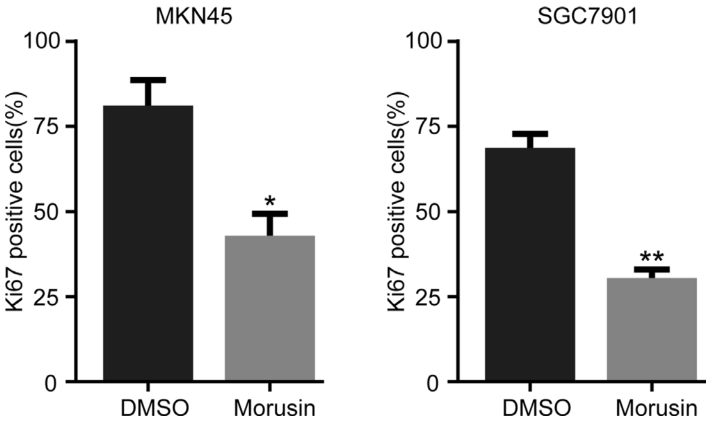

Supplementary Figure 3: Morusin inhibits the expression of Ki67 in xenograft tumor. The histogram demonstrates the results of quantifying the rate of Ki67-positive cells. All data were analyzed using 2-tailed Student's tests. Error bars, *$P < 0.05$, **$P < 0.01$, and ***$P < 0.001$.

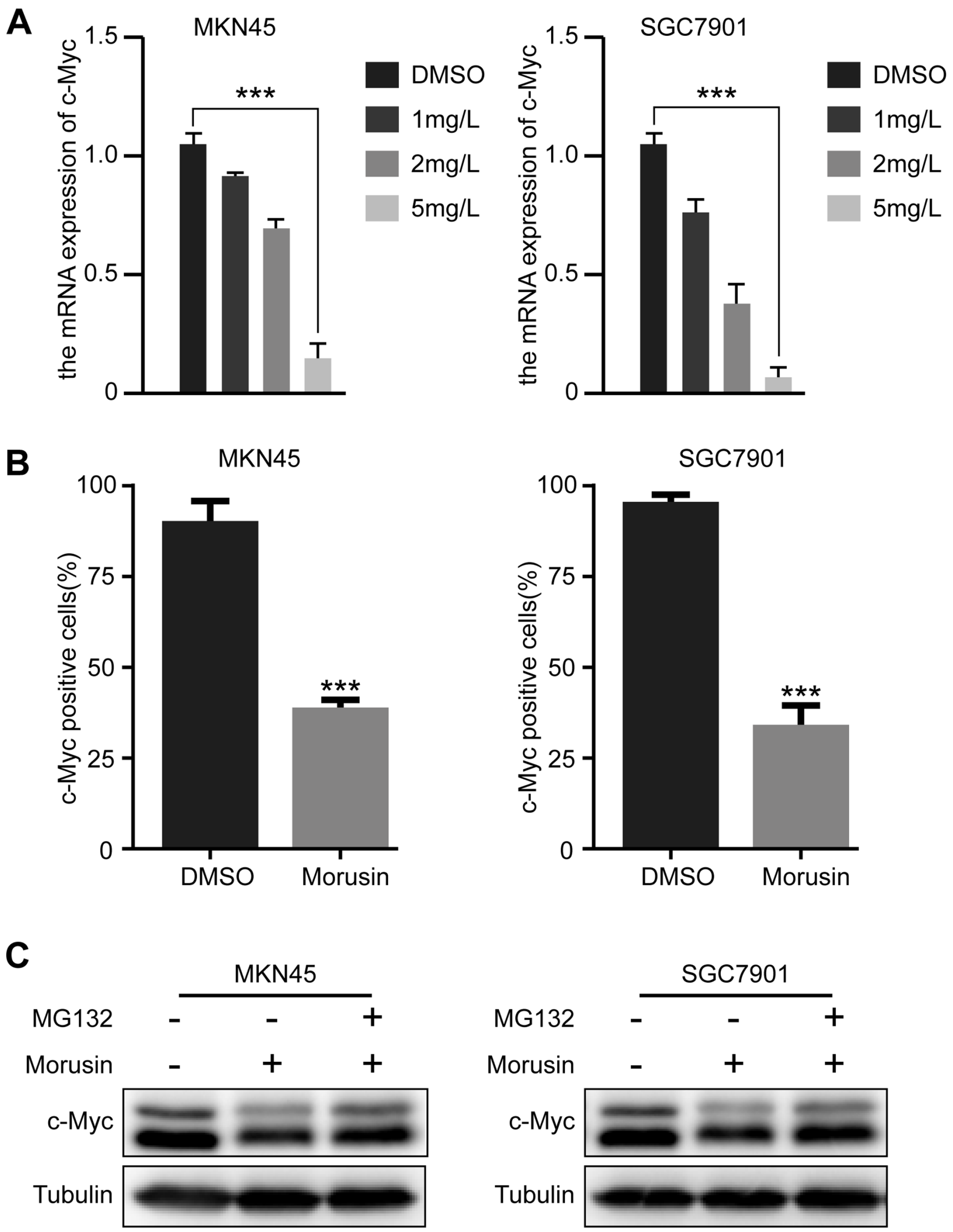

Supplementary Figure 4: Morusin inhibits the expression level of c-Myc. (**A**) After cells were treated with morusin at different concentrations, the expression levels of c-Myc were determined using qRT-PCR assays. (**B**) The histogram demonstrates the results of quantifying the rate of c-Myc -positive cells. (**C**) The expression level of c-Myc was determined using western blot analysis after cells were treated with morusin or MG132. Tubulin was used as a loading control. All data were analyzed using 2-tailed Student's tests. Error bars, *$P < 0.05$, **$P < 0.01$, and ***$P < 0.001$.

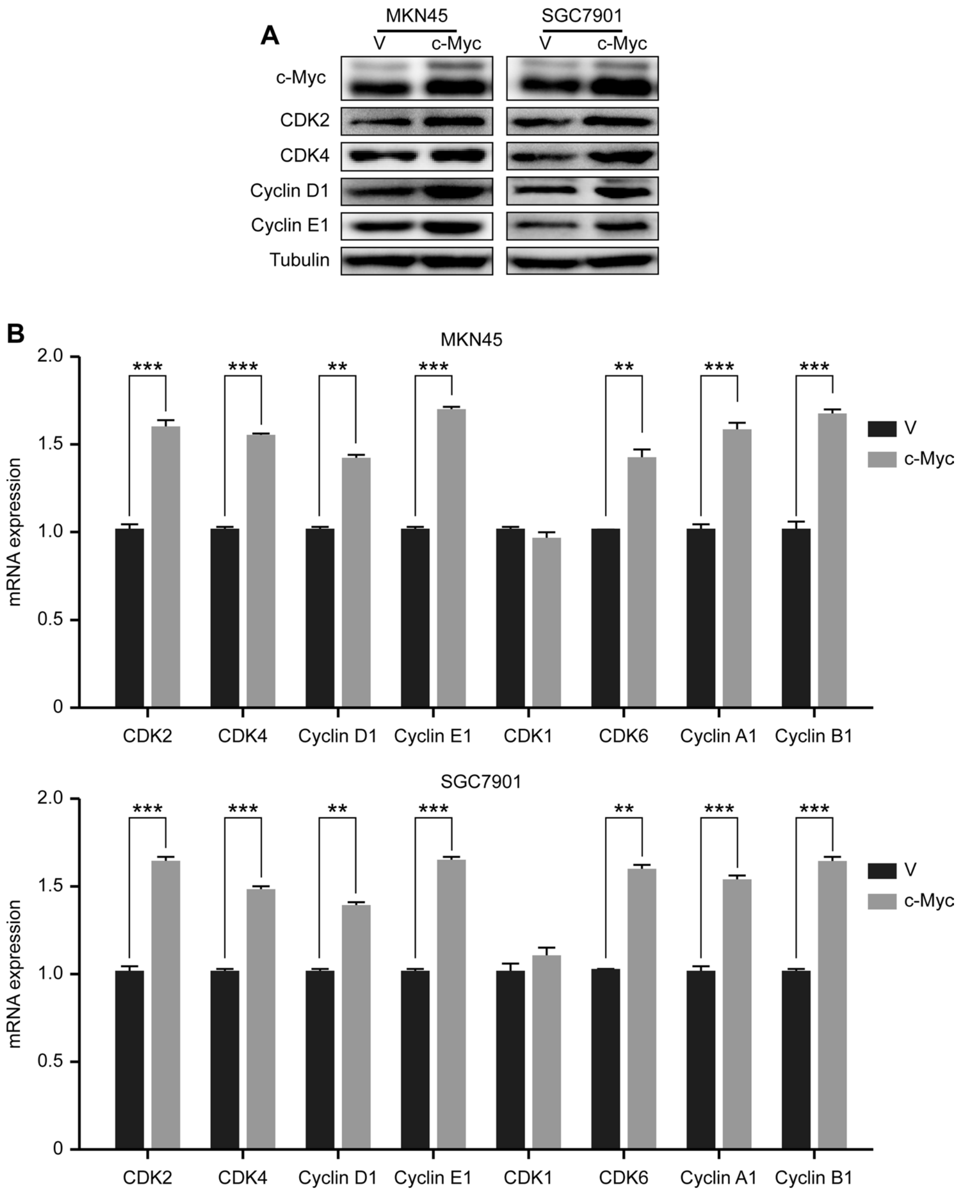

Supplementary Figure 5: c-Myc induces CDKs and Cyclins up-regulation. (**A**, **B**) After c-Myc overexpressed, the expression levels of CDKs and Cyclins were determined using western blot analysis (A) and qRT-PCR assays (B). All data were analyzed using 2-tailed Student's tests. Error bars, *$P < 0.05$, **$P < 0.01$, and ***$P < 0.001$.

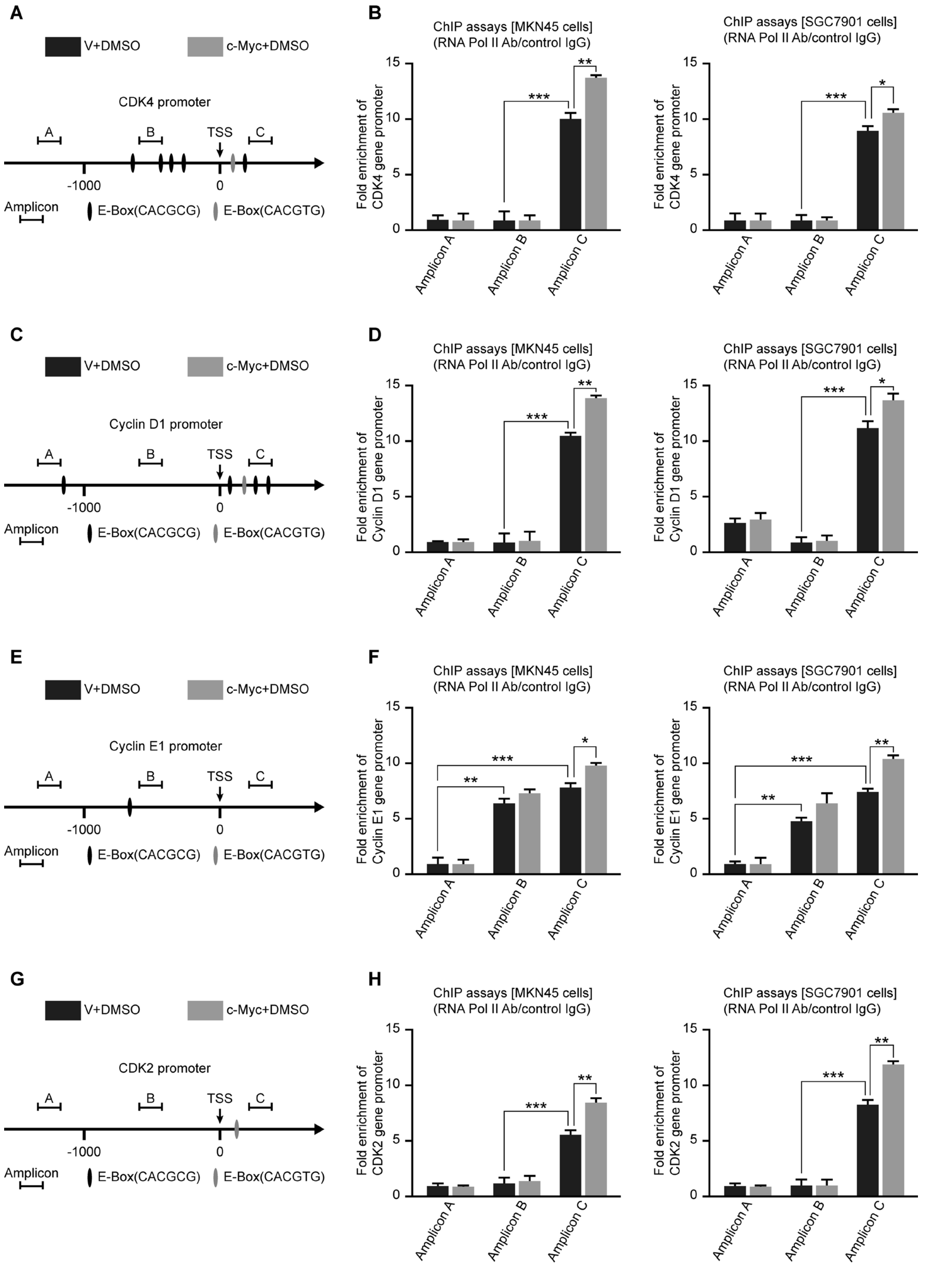

Supplementary Figure 6: c-Myc increases binding of RNA Pol II to CDKs and Cyclins promoters. (**A**, **C**, **E**, **G**) Schematic representation of the CDK2, CDK4, Cyclin D1 and Cyclin E1 promoters, as indicated. TSS, transcription start site. (**B**, **D**, **F**, **H**) ChIP assays were performed using MKN45 and SGC7901 cells and RNA Pol II antibody. This was followed by qRT-PCR using primers targeting the CDK2, CDK4, Cyclin D1 and Cyclin E1 promoters, as indicated. Rabbit IgG was used as the negative control. All data were analyzed using 2-tailed Student's tests. Error bars, $*P < 0.05$, $**P < 0.01$, and $***P < 0.001$.

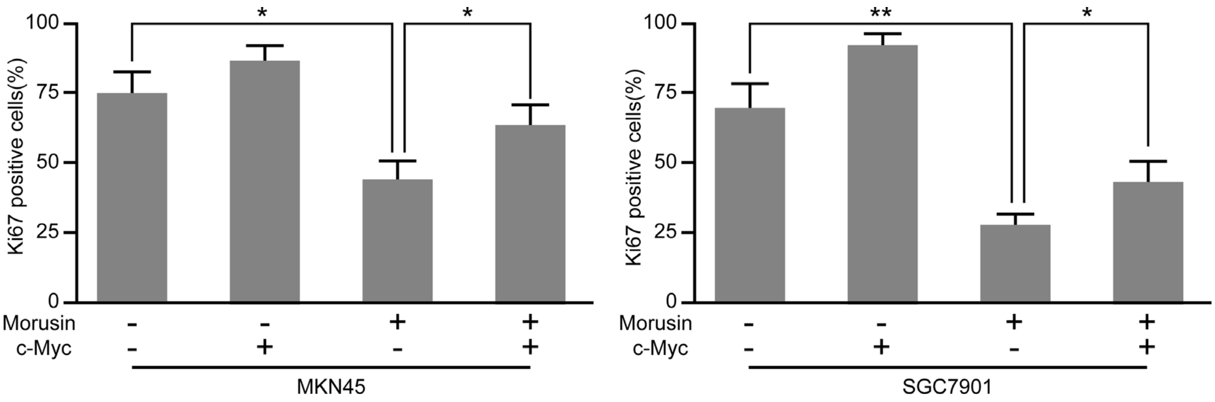

Supplementary Figure 7: Overexpressing c-Myc rescues morusin-induced Ki67 down-regulated. The histogram demonstrates the results of quantifying the rate of Ki67-positive cells. All data were analyzed using 2-tailed Student's tests. Error bars, $*P < 0.05$, $**P < 0.01$, and $***P < 0.001$.

Supplementary Table 1: The sequence of primers used in ChIP assays

Gene	Amplicon	Primer
CDK2	A	F: TCTATCCTTCTGCCCCTCCA
		R: GGCTGTGATAGGTGCTTTGCT
	B	F: ACATTGCTTTCCCATCCTGC
		R: AGTCGTTGCCTCTGCCTCATA
	C	F: GGAAAACTTTCTTCCCAAACTGAG
		R: GGTTCCCACATAACTCCACTCC
CDK4	A	F: GTAAGGAATGGAATCGGATTGTC
		R: TCTCCATAAAAGGAAAGGGGTAAG
	B	F: CCCCTTAGTCAGTTCAGTCCTTG
		R: AATACAGATGCCTCCTCTTTCCAT
	C	F: CCCCATTGTTGGCTACATCTT
		R: ACATTTTACTGAGTGCTTGCTTCC
CyclinD1	A	F: CTGCCAGGAGCAGATCGAA
		R: CAACCAACAACAAGGAGAATGAAG
	B	F: CAACCAACAACAAGGAGAATGAAG
		R: GCTATTTCCTACACCTATTGGACTGA
	C	F: CAAGCTGCCGAACCAAAAG
		R: GAGGTAAGCGTGAGCCGTGT
CyclinE1	A	F: CCTAACGGGCACAGGATGG
		R: GCGTAAAAGAAGGCTCTGGAAT
	B	F: ACGACCCAATGCACTGACG
		R: CTGTGCCTTGGCCTAGAACC
	C	F: TAATGCAGCCACAGCCCATA
		R: GCCAGTCAGGAACACCAAGC
